# 332. Factors Associated with Readmission in Patients with End-Stage Renal Disease on Hemodialysis Discharged on Outpatient Parenteral Antimicrobial Therapy

**DOI:** 10.1093/ofid/ofad500.403

**Published:** 2023-11-27

**Authors:** Ghaleb Ismail, Nancy MacDonald, Rachel M Kenney, Surafel MULUGETA, Suzette Gendjar, Chantale Daifi

**Affiliations:** Henry Ford Health System, Dearborn, Michigan; Henry Ford Hospital, Washington Twp, Michigan; Henry Ford Hospital, Washington Twp, Michigan; Henry Ford Health, Detroit, Michigan; Henry Ford Health System, Dearborn, Michigan; Henry Ford Health System, Dearborn, Michigan

## Abstract

**Background:**

Outpatient parenteral antimicrobial therapy (OPAT) is a popular option for patients who require extended treatment with antimicrobials. OPAT in patients with end stage renal disease (ESRD) on hemodialysis (HD) has unique considerations and regimens that facilitate vein preservation as a priority. This study aimed to characterize patients with ESRD on HD discharged on OPAT and identify factors associated with poor outcomes.

**Methods:**

This was an IRB–approved retrospective cohort of patients ≥ 18 years with ESRD on HD who were discharged between 1/1/20 and 8/30/22 with at least one week of OPAT. Enrolled patients were divided into two equal groups depending on their 60-day readmission status. Patients were excluded if they were admitted on OPAT prior to admission or expired within 30 days of the initial OPAT discharge, incarcerated, or if the discharge was self-directed. To identify risk factors for readmission, patients who had an unplanned all-cause 30-day readmission were compared with those not readmitted. Other safety measures collected include transitions of care process measures, pharmacist collaboration with patient education and follow-up monitoring.

**Results:**

162 patients (67 females, 95 males; median age 59 years) were included. The most common reason for OPAT was bloodstream infection (51%). Vancomycin was the most prescribed (50%). Nine patients in the readmitted group reported adverse events compared to 31 in the readmitted group (p = < 0.001). Patients in the non-readmitted were statistically more likely to have a pharmacist infection treatment plan note prior to discharge (p = 0.036) and statistically more likely to attend infectious disease (ID) follow-up appointments (p = 0.001). A multivariable regression identified that being female, having diabetes mellitus, or congestive heart failure are associated with increased risk of unfavorable outcomes (Adjusted odd ratio = 3.352, 1.75, and 1.671 respectively).

**Conclusion:**

This study suggests that OPAT optimization and patient education by a pharmacist, and attending follow-up ID appointments reduced the risk of readmission for patients with ESRD on HD.

**Disclosures:**

**All Authors**: No reported disclosures

